# Combined MicroRNA-340 and ROCK1 mRNA Profiling Predicts Tumor Progression and Prognosis in Pediatric Osteosarcoma

**DOI:** 10.3390/ijms15010560

**Published:** 2014-01-06

**Authors:** Haiqing Cai, Lijun Lin, Haikang Cai, Mingjie Tang, Zhigang Wang

**Affiliations:** 1 Pediatric Orthopedic Department, Shanghai Children’s Medical Center, Shanghai Jiaotong University School of Medicine, Shanghai 200031, China; E-Mail: gangqing@vip.sina.com; 2 Orthopedics Department, Zhujiang Hospital, the Southern Medical University, Guangzhou 510282, China; E-Mail: linlijun1@126.com; 3 Orthopaedics Department, Xuhui Central Hospital, Shanghai 200233, China; E-Mail: caihaikang@163.com; 4 Orthopaedics Department, Shanghai Sixth People’s Hospital, Shanghai Jiaotong University, Shanghai Institute for Microsurgery of Extremities, Shanghai 200233, China; E-Mail: 13311828153@189.cn

**Keywords:** pediatric osteosarcoma, microRNA-340, ROCK1, clinicopathologic features, overall survival, progression-free survival

## Abstract

To investigate the association of combined microRNA-340 (miR-340) and ROCK1 mRNA profiling with clinicopathologic features and prognosis in pediatric patients with osteosarcoma. Quantitative real-time reverse transcriptase-polymerase chain reaction analysis was performed to detect expression levels of miR-340 and ROCK1 mRNA in cancerous and noncancerous bone tissues from 92 children treated for primary osteosarcomas. Compared with noncancerous bone tissues, the expression levels of miR-340 and ROCK1 mRNA were, respectively, downregulated and upregulated in osteosarcoma tissues (both *p* < 0.001), which was consistent with the results of *in situ* hybridization and immunohistochemistry analysis. The downregulation of miR-340 was negatively correlated with the upregulation of ROCK1 mRNA in osteosarcoma tissues (*r* = −0.78, *p* = 0.001). In addition, the combined miR-340 downregulation and ROCK1 upregulation (miR-340-low/ROCK1-high) occurred more frequently in osteosarcoma tissues with positive metastasis (*p* < 0.001) and poor response to pre-operative chemotherapy (*p* = 0.002). Moreover, miR-340-low/ROCK1-high expression was significantly associated with both shortest overall survival (*p* < 0.001) and progression-free survival (*p* < 0.001). Multivariate analysis further confirmed that miR-340-low/ROCK1-high expression was an independent prognostic factor of unfavorable survival in pediatric osteosarcoma (for overall survival: *p* = 0.006, for progression-free survival: *p* = 0.008). Our data offer convincing evidence, for the first time, that the combined miR-340 downregulation and ROCK1 upregulation may be linked to tumor progression and adverse prognosis in pediatric osteosarcoma.

## 1. Introduction

Osteosarcoma is the most common primary malignant bone tumor predominantly seen in children and adolescents [1]. Although its incidence is relative low (approximately four to five cases per million), osteosarcoma often arises from the metaphysis of the long bones [2]. Treatment of this tumor is usually a combination of surgery and chemotherapy. While the five-year overall survival rate of patients with osteosarcoma has dramatically improved, to approximately 60%–70% [3], there are a large number of patients who respond poorly to chemotherapy and have a high risk of local relapse or distant metastasis, even after curative resection of the primary tumor and intensive chemotherapy [4]. To address this problem, much work has been performed in an attempt to screen markers with therapeutic implications. Studies of molecular biology have identified several molecules, tumor pathways, and specific mediators of osteosarcoma progression [5,6]. However, the tumorigenesis of osteosarcoma has not been fully elucidated. Thus, it is imperative to identify tumor-derived factors that are associated with the early diagnosis and the prognosis may be helpful to detect chemotherapy-resistant tumors and to generate a modified treatment regimen for this deadly disease.

MicroRNAs (miRNAs) represents an abundant class of small (usually 18–25 nucleotides in length) and noncoding RNAs that often negatively modulate gene expression at the post-transcriptional level via incomplete or complete complementary binding to target sequences within the 3′ untranslated region of mRNA [7,8]. Since the discovery of the first miRNA, lin-4, in *C. elegans*, in 1993, it has been estimated that as many as 1000 miRNAs exist in the human genome [9]. About 10%–30% of genes [7], especially those related to signal transduction, may be regulated by miRNAs. Multiple studies have demonstrated that miRNAs are involved in the regulation of various cellular processes, such as cell cycle, apoptosis, hematopoietic cell differentiation, metabolism, neural development, and metastasis [10,11]. Aberrant and absent expression of miRNAs are usually associated with pathophysiology disorders, especially various human malignancies. miRNAs may function either as tumor suppressors or oncogenes depending on specific tumor types. In osteosarcoma, Jones *et al.* [12] identified for the first time several miRNA signatures, including high expression of miR-181a, miR-181b, and miR-181c, as well as reduced expression of miR-16, miR-29b, and miR-142-5p to reflect the tumor pathogenesis, our previous study found that the overexpressions of miR-210 and miR-214 were both strongly associated with tumor aggressive progression of pediatric osteosarcoma and could help prognostic screening of patients with this malignancy [13,14], Novello *et al.* [15] indicated that the expression of miR-1 and miR-133b may control cell proliferation of osteosarcoma cells. These findings suggested that miRNAs play a significant role in regulating globally molecular signaling networks during the tumorigenesis of osteosarcoma.

MicroRNA-340 (miR-340) has been identified as tumor suppressor in various human cancers, including breast cancer, colorectal cancer, and gastric cancer [16–18]. More interestingly, Zhou *et al.* [19] reported that miR-340 may inhibit osteosarcoma tumor growth and metastasis by directly targeting ROCK1, which is a GTP-dependent serine/threonine protein kinase interacting with the Rho G-protein through its Rho-binding domain, thereby mediating Rho signaling [20,21]. At present, little research has focused on the prognostic values of miR-340 and ROCK1 in osteosarcoma. Accumulating studies indicated that the combined miRNA and mRNA expression profiling may potentially provide diagnostic and prognostic information for various human cancers. On this basis, we hypothesized that the combined dysregulation of miR-340 and its target mRNA ROCK1 might be associated with tumor progression and prognosis in patients with osteosarcoma. In the current study, we performed quantitative real-time reverse transcriptase-polymerase chain reaction (qRT-PCR), *in situ* hybridization and immunohistochemistry analyses to respectively detect expression levels of miR-340 and ROCK1 in cancerous and noncancerous bone tissues from 92 children treated for primary osteosarcomas. We also evaluated the clinical significance of miR-340 and ROCK1 dysregulation in pediatric osteosarcomas.

## 2. Results

### 2.1. Expression Patterns of miR-340 and ROCK1 in Pediatric Osteosarcoma Tissues

**Figure 1.**
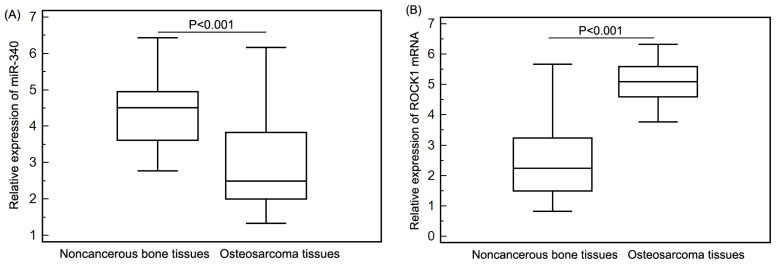
miR-340 (**A**) and ROCK1 mRNA (**B**) expressions in 92 pairs of osteosarcoma and corresponding noncancerous bone tissues were respectively detected by qRT-PCR assay. Compared with noncancerous bone tissues, miR-340 expression was significantly decreased in osteosarcoma tissues (mean ± SD, osteosarcoma *vs.* noncancerous bone: 2.88 ± 1.11 *vs.* 4.46 ± 0.94, *p* < 0.001), while ROCK1 expression was significantly increased in osteosarcoma tissues (mean ± SD, osteosarcoma *vs.* noncancerous bone: 5.11 ± 0.58 *vs.* 2.49 ± 1.11, *p* < 0.001).

**Figure 2.**
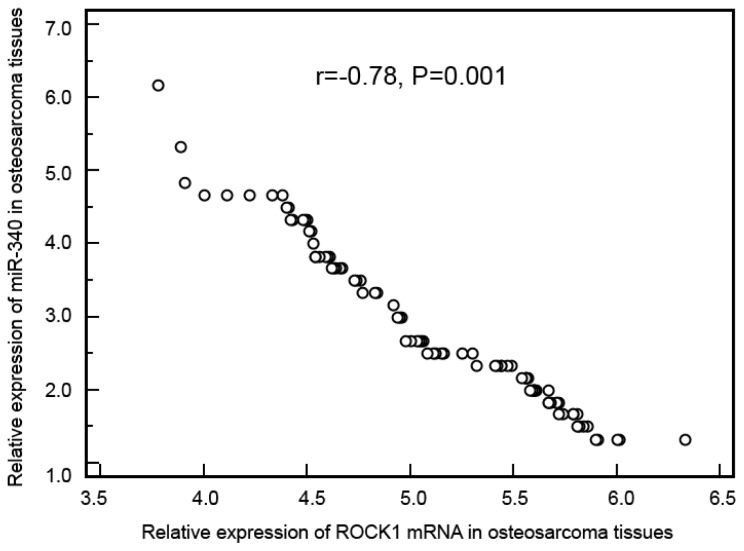
Scatter diagram for the correlation analysis of miR-340 expression and ROCK1 mRNA expression in osteosarcoma tissues.

The expression levels of miR-340 and ROCK1 mRNA in osteosarcoma and corresponding noncancerous bone biopsy samples were detected by qRT-PCR, and respectively normalized to RNU6B and β-actin. The qRT-PCR assays for a particular gene were undertaken at the same time for all samples under identical conditions, in triplicate. Compared with noncancerous bone tissues, miR-340 expression was significantly decreased in osteosarcoma tissues (mean ± SD, osteosarcoma *vs.* noncancerous bone: 2.88 ± 1.11 *vs.* 4.46 ± 0.94, *p* < 0.001, Figure 1A), while ROCK1 expression was significantly increased in osteosarcoma tissues (mean ± SD, osteosarcoma *vs.* noncancerous bone: 5.11 ± 0.58 *vs.* 2.49 ± 1.11, *p* < 0.001, Figure 1B). In addition, the median values of miR-340 (2.88) and ROCK1 mRNA (5.10) expression levels in all osteosarcoma tissues were used as cutoff points to classify 92 patients with osteosarcomas into miR-340-low (*n* = 50), miR-340-high (*n* = 42), ROCK1-low (*n* = 40) and ROCK1-high (*n* = 52) expression groups. On this basis, eight (8.70%) cases were both low expression of miR-340 and ROCK1, 10 (10.87%) cases were both high expression of miR-340 and ROCK1, 32 (34.78%) cases were miR-340-high and ROCK1-low expression, and 42 (45.65%) cases were miR-340-low and ROCK1-high expression. As determined by Spearman’s correlation, the downregulation of miR-340 was negatively correlated with the upregulation of ROCK1 mRNA in osteosarcoma tissues (*r* = −0.78, *p* = 0.001, Figure 2).

**Figure 3.**
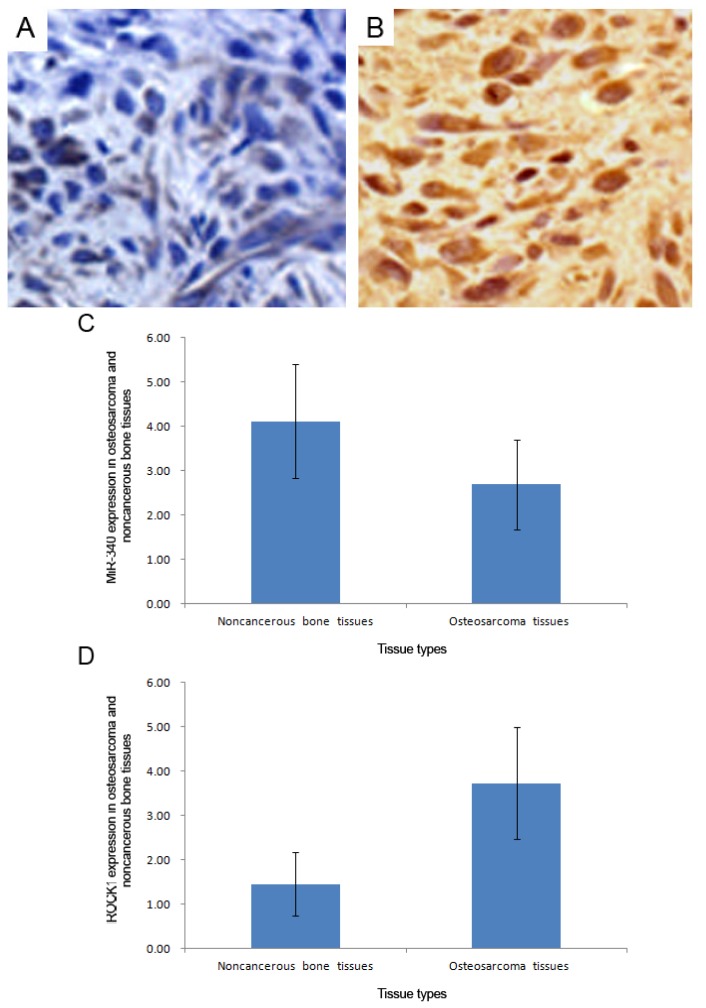
Representative *in situ* hybridization (**A**) and immunohistochemistry (**B**) images respectively for miR-340 and ROCK1 protein expression in osteosarcoma and corresponding noncancerous bone tissues (Original magnification ×200); Compared with the corresponding noncancerous bone tissues, the expression level of miR-340 (mean ± S.D.: 2.69 ± 1.01 *vs.* 4.11 ± 1.36, *p* < 0.001) (**C**) was significantly decreased, while the expression level of ROCK1 protein (mean ± S.D.: 1.40 ± 0.83 *vs.* 3.74 ± 1.25, *p* < 0.001) (**D**) was significantly increased in osteosarcoma tissues.

*In situ* hybridization and immunohistochemistry analysis respectively showed that the positive stainings of miR-340 (Figure 3A) and ROCK1 protein (Figure 3B) were both localized in cytoplasm of tumor cells in osteosarcoma tissues. Compared with the corresponding noncancerous bone tissues, the expression level of miR-340 (mean ± S.D.: 2.69 ± 1.01 *vs.* 4.11 ± 1.36, *p* < 0.001, Figure 3C) was significantly decreased, while the expression level of ROCK1 protein (mean ± S.D.: 1.40 ± 0.83 *vs.* 3.74 ± 1.25, *p* < 0.001, Figure 3D) was significantly increased in osteosarcoma tissues, which was similar with the results of qRT-PCR.

### 2.2. miR-340 Downregulation and ROCK1 Upregulation Associate with Aggressive Clinicopathological Features of Pediatric Osteosarcoma

Table 1 summarized the associations of miR-340 and ROCK1 mRNA expression with various clinicopathological parameters of osteosarcoma tissues. The downregulation of miR-340 was significantly associated with tumors with large tumor size (*p* = 0.02), positive metastasis (*p* = 0.001) and poor response to pre-operative chemotherapy (*p* = 0.002). In addition, the upregulation of ROCK1 mRNA expression was significantly associated with patients with positive metastasis (*p* = 0.001) and poor response to pre-operative chemotherapy (*p* = 0.002). More interestingly, the combined miR-340 downregulation and ROCK1 upregulation (miR-340-low/ROCK1-high) occurred more frequently in osteosarcoma tissues with positive metastasis (*p* < 0.001) and poor response to pre-operative chemotherapy (*p* = 0.002). However, there was no significant association between miR-340 or ROCK1 mRNA expression and other clinicopathological parameters, including patients’ gender and age at diagnosis, localization of the primary tumor, pathological facture, subtype of osteosarcoma, and chemotherapy (all *p* > 0.05, Table 1).

### 2.3. miR-340 Downregulation and ROCK1 Upregulation Confer Poor Prognosis in Pediatric Osteosarcoma

**Figure 4.**
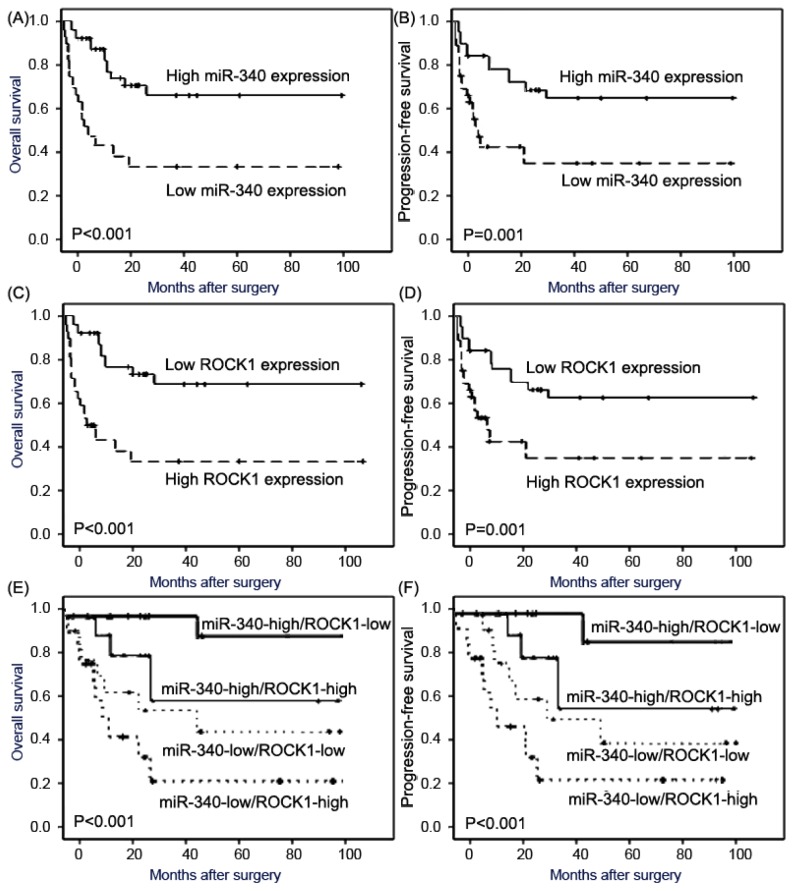
Kaplan-Meier survival curves for osteosarcoma patients with high and low expression of miR-340 [(**A**) for overall survival, (**B**) for progression-free survival], ROCK1 mRAN [(**C**) for overall survival, (**D**) for progression-free survival], and concomitant miR-340 and ROCK1 [miR-340/ROCK1, (**E**) for overall survival, (**F**) for progression-free survival].

Using Kaplan-Meier method and log-rank test, the overall survival (OS, Figure 4A, both *p* < 0.001) and progression-free survival (PFS, Figure 4B, both *p* = 0.001) of pediatric osteosarcoma patients with low miR-340 expression or high ROCK1 expression were both significantly shorter than those with high miR-340 expression or low ROCK1 expression. In addition, the association between combined expression status of miR-340/ROCK1 and the prognosis of patients with osteosarcomas was also tested by the Kaplan-Meier method. The Chi-square value by Log Rank (Mantel-Cox) indicated a significant difference among different groups with regard to the combined expression status of miR-340/ROCK1 (Figure 4C). The results of pairwise comparisons showed that the statistically significant differences of OS and PFS existed between miR-340-low/ROCK1-high patients and any of other three groups (both *p* < 0.001). In all four groups, miR-340-low/ROCK1-high patients had the shortest OS and PFS. The survival benefits were also found in those with smaller tumor size (*p* = 0.008 and 0.01, respectively), without metastasis (*p* < 0.001 and 0.001, respectively), and better response to pre-operative chemotherapy (both *p* = 0.01) for OS and PFS.

Cox proportional hazard model confirmed that miR-340 expression (for OS: RR 6.2, 95% CI, 1.4–13.9, *p* = 0.006, for PFS: RR 4.5, 95% CI, 1.0–9.2, *p* = 0.01), ROCK1 expression (for OS: RR 5.8, 95% CI, 1.2–12.6, *p* = 0.008, for PFS: RR 3.2, 95% CI, 1.0–8.8, *p* = 0.02), miR-340/ROCK1 expression (for OS: RR 7.6, 95% CI, 1.5–16.2, *p* = 0.002, for PFS: RR 6.5, 95% CI, 1.0–8.2, *p* = 0.005), tumor size (for OS: RR 3.5, 95% CI, 1.0–8.4, *p* = 0.01, for PFS: RR 2.9, 95% CI, 0.8–7.6, *p* = 0.02), metastasis status (for OS: RR 4.2, 95% CI, 1.6–10.3, *p* = 0.006, for PFS: RR 3.9, 95% CI, 1.3–9.6, *p* = 0.008), and .response to pre-operative chemotherapy (for OS: RR 2.5, 95% CI, 0.9–7.3, *p* = 0.02, for PFS: RR 2.1, 95% CI, 0.6–6.6, *p* = 0.03) were all independent prognostic factors of unfavorable survival in pediatric osteosarcoma (Table 2).

## 3. Discussion

Accumulating evidence has shown that combined miRNA and mRNA profiling may be involved in multiple processes in cancer development and progression. In the current study, we firstly observed that miR-340 and ROCK1 mRNA expression levels were respectively decreased and increased in pediatric osteosarcoma tissues compared with noncancerous bone tissues, which was consistent with the results of *in situ* hybridization and immunohistochemistry analysis. In addition, the downregulation of miR-340 and the upregulation of ROCK1 mRNA in osteosarcoma tissues were both significantly correlated with aggressive clinicopathological features. Notably, the combined miR-340 downregulation and ROCK1 mRNA upregulation was significantly associated with the presence of tumor metastasis and the poor response to pre-operative chemotherapy. Moreover, the results of Kaplan-Meier analyses shown that osteosarcoma tissues with high miR-340 expression, low ROCK1 mRNA expression and the combined miR-340 downregulation and ROCK1 mRNA upregulation tend to have shorter overall survival and progression-free survival. Finally, the multivariate analysis clearly indicated that high miR-340 expression, low ROCK1 mRNA expression and the combined miR-340 downregulation and ROCK1 mRNA upregulation may be all considered as prognostic factors in pediatric osteosarcoma for decreased survival and a greater probability of disease progression, regardless of oncological treatment.

Recent studies have demonstrated that miR-340 expression is suppressed in malignant tissues compared with the normal and benign tissue samples and that lost miR-340 expression may contribute to tumorigenesis and tumor progression. For example, Sun *et al.* [16] indicated that miR-340 expression inhibited the growth of colorectal cancer cells and was associated with poor prognosis of colorectal cancer, and Wu *et al.* [17] reported that loss of miR-340 expression was associated with lymph node metastasis, high tumor histological grade, clinical stage, and shorter overall survival of breast cancer. Especially, Zhou *et al.* [19] found that the overexpression of miR-340 in osteosarcoma cell lines significantly inhibited cell proliferation, migration, and invasion *in vitro*, and tumor growth and metastasis in a xenograft mouse model. In line with these findings, our data in the current study showed that the expression levels of miR-340 in human osteosarcoma tissues were dramatically reduced and the downregulated miR-340 expression was closely correlated with advanced tumor progression and poor prognosis in patients with osteosarcoma, suggesting miR-340 may function as a tumor suppressor in this malignancy.

Rho-associated serine-threonine protein kinase (ROCK) is one of the best characterized downstream effectors of Rho [20]. Two ROCK isoforms have been identified: ROCK1/ROKβ and ROCK2/ROKα, which function as key downstream effectors of the RhoA small GTPase [21]. ROCKs are activated when they selectively bind to the active GTP-bound form of Rho. Activated ROCKs interact with the actin cytoskeleton to promote stress-fiber formation and assembly of focal contacts [22]. ROCKs regulate diverse cellular processes, such as actomyosin contractility, focal adhesion assembly, cytokinesis, and cell proliferation [23]. There is considerable and growing evidence for the importance of the ROCKs in oncogenesis. Especially regarding ROCK1, Vigil *et al.* [24] demonstrated that ROCK1 was required for non-small cell lung cancer anchorage-independent growth and invasion, Wu *et al.* [25] found that the positive expression rates of ROCK1 expression in normal tissue, dysplasia and gastric carcinoma showed an increasing trend and were correlated with tumor lymph node metastasis and TNM stage, Majid *et al.* [26] showed that oncogene ROCK1 may act as a direct target of tumor suppressor miR-1280 in bladder cancer. In osteosarcoma, and Liu *et al.* [27] indicated that the knockdown of ROCK1 could inhibit proliferation and induces apoptosis in osteosarcoma cell lines and high levels of ROCK1 were associated with poor outcomes in clinical osteosarcoma, which was consistent with our findings.

More importantly, we found that the combined miR-340 and ROCK1 mRNA expression profiling may be more significantly associated with tumor aggressiveness of pediatric osteosarcoma than the abnormal expression of miR-340 or ROCK1 mRNA alone. In agreement with the findings of Zhou *et al.* [19] the downregulation of miR-340 and the upregulation of its target gene ROCK1 may predict advanced tumor progression and unfavorable prognosis in patients with osteosarcoma.

## 4. Materials and Methods

### 4.1. Patients and Tissue Samples

This study was approved by the Research Ethics Committee of Shanghai Children’s Medical Center Affiliated to Shanghai Jiaotong University School of Medicine, Zhujiang Hospital, Xuhui Central Hospital, and Shanghai Sixth People’s Hospital, China. Written informed consent was obtained from all of the patients. All specimens were handled and made anonymous according to the ethical and legal standards.

For qRT-PCR analysis, 92 pediatric patients with osteosarcomas (age range: 4–20 years, median 13 years) and corresponding noncancerous bone tissue samples from the same specimens were collected from Shanghai Children’s Medical Center Affiliated to Shanghai Jiaotong University School of Medicine, Zhujiang Hospital, Xuhui Central Hospital and Shanghai Sixth People’s Hospital, China, from 1999 to 2008. After establishing the diagnosis, all patients were treated with a preoperative chemotherapy, lasting four months, using either the combination of an anthracycline (doxorubicin) and high-dose methotrexate or the combination of etoposide, ifosfamide, and high-dose methotrexate. The postoperative treatment was determined by the histologic system established by Huvos *et al.* [22]. The following clinical parameters were analyzed: age, gender, site of tumor, tumor size, presence of pathological fracture and distant metastasis, histological subtype of osteosarcoma, type of surgery, and histological response to pre-operative chemotherapy. The Huvos grading system was used to rate the level of tumor necrosis following preoperative chemotherapy [23]. The good responders were defined as patients whose tumors had ≥90% necrosis in response to preoperative chemotherapy as determined by histologic examination at the time of definitive surgery and poor responders had <90% necrosis. The clinicopathological information of the patients is shown in Table 1. Tumor biopsies were collected before neoadjuvant therapy and were fresh frozen, stored at −80 ºC, and histologically characterized by the pathologist.

All 92 pediatric patients with osteosarcoma received follow-up. The median follow-up was 82 months (range: 10–133 months). During the follow-up period, 36 patients (36/92, 39.1%) died of disease. Metastases developed in 31 patients at a mean of 16.2 months (range 5–49 months) after the original diagnosis. The median overall survival and progression-free survival (PFS) of patients was 30 months (95% confidence interval [CI], 26.4–42.7 months) and 22 months (95% CI, 18.3–32.9 months), respectively.

### 4.2. RNA Extraction

Total RNA and small RNA from fresh osteosarcoma and corresponding noncancerous tissues were, respectively, extracted using an RNeasy Mini Kit (Qiagen, Valencia, CA, USA) and a mirVana miRNA Isolation Kit (Ambion, Austin, TX, USA), according to the manufacture’s instruction.

### 4.3. miRNA and mRNA qRT-PCR Assay

The expression levels of miR-340 and ROCK1 in osteosarcoma and corresponding noncancerous tissues were detected by qRT-PCR assay. Total RNA (1 μg) and small RNA (10 ng) were, respectively, synthesized with the ReverTra Ace qRT Kit (Toyobo, Osaka, Japan) and TaqMan MicroRNA RT Kit (Applied Biosystems, Foster City, CA, USA). Real-time PCR was performed in ABI 7500 (Applied Biosystems). U6B and human β-actin were amplified as endogenous controls. The sequences of the primers were as follows: miR-340 forward, 5′-GCG GTT ATA AAG CAA TGA GA-3′, reverse, 5′-GTG CGT GTC GTG GAG TCG-3′, U6 forward 5′-CTC GCT TCG GCA GCA CA-3′ and reverse 5′-AAC GCT TCA CGA ATT TGC GT-3′, human ROCK1 forward 5′-AGG AAG GCG GAC ATA TTA GTC CCT-3′ and reverse 5′-AGA CGA TAG TTG GGT CCC GGC-3′, human β-actin forward 5′-TGA CGT GGA CAT CCG CAA AG-3′ and reverse 5′-CTG GAA GGT GGA CAG CGA GG -3′. The PCR condition was according to the manufacturer’s instructions. The 2^−ΔΔ^*^C^*^t^ relative quantification method [24] was used to calculate relative miRNA and mRNA expression. The levels of miR-340 and ROCK1 expression were measured using *C*t (threshold cycle). The *C*t is the fractional cycle number at which the fluorescence of each sample passes the fixed threshold. The ΔΔ*C*t method for relative quantitation of gene expression was used to determine miRNA and mRNA expression levels. The Δ*C*t was calculated by subtracting the *C*t of RNU6B or β-actin from the Ct of miR-340 or ROCK1. The ΔΔ*C*t was calculated by subtracting the Δ*C*t of the reference sample from the Δ*C*t of each sample. Fold change was generated by using the equation 2^−ΔΔ^*^C^*^t^. A pool of two normal bone tissues was used for the standard curve calculation and as reference sample for the Δ*C*t.

### 4.4. *In Situ* Hybridization

*In situ* hybridization was performed to detect the expression level and subcellular localization of miR-340 in osteosarcoma and corresponding noncancerous tissues. Briefly, the tissue slides were hybridized with 200 nM of 5′-digoxigenin (DIG) LNA-modified-miR-340 (Exiqon, Copenhagen, Denmark) using IsHyb *in situ* Hybridization kit (Biochain, Eureka Drive, Newark, CA, USA), according to manufacturer’s instructions.

### 4.5. Immunohistochemistry Analysis

Immunohistochemical staining was carried out to detect the expression level and subcellular localization of ROCK1 protein in osteosarcoma and corresponding noncancerous tissues. Briefly, tissue slides were incubated at 4 ºC, overnight with rabbit monoclonal ROCK1 antibody (1: 100; Abcam, Cambridge, UK). Secondary antibody for the detection of primary antibody: anti-rabbit IgG (#sc-3739, Santa Cruz Biotechnology, Inc., Santa Cruz, CA, USA). The negative controls were treated identically, but without the primary antibody. The positive ROCK1 expression, confirmed by Western blotting, was used as positive controls for immunostaining.

### 4.6. Evaluation of *in Situ* Hybridization and Immunostaining

*In situ* hybridization and immunostaining results were scored by two independent experienced pathologists, who were blinded to the clinicopathological data and clinical outcomes of the patients. The scores of the two pathologists were compared and any discrepant scores were re-examined by both pathologists to reach a consensus score. The number of positive-staining cells, in ten representative microscopic fields, was counted and the percentage of positive cells was calculated. The percentage scoring of positive tumor cells was as follows: 0 (0%), 1 (1%–10%), 2 (11%–50%) and 3 (>50%). The staining intensity was visually scored and stratified as follows: 0 (negative), 1 (weak), 2 (moderate), and 3 (strong). A final score was obtained for each case by multiplying the percentage and the intensity score. Therefore, tumors with a multiplied score exceeding median of total score for miR-340 or ROCK1 protein were deemed to be low expressions of miR-340 or ROCK1 protein; all other scores were considered to be high expressions of miR-340 or ROCK1 protein.

### 4.7. Statistical Analysis

The software of SPSS version13.0 for Windows (SPSS Inc., Chicago, IL, USA) and SAS 9.1 (SAS Institute, Cary, NC, USA) was used for statistical analysis. Continuous variables were expressed as *X̄* ± *s*. The paired t test was used to evaluate differences of miR-340 or ROCK1 expression levels in osteosarcoma and corresponding noncancerous bone tissues. The Chi-square test was used to show differences of categorical variables. Patient survival and their differences were determined by Kaplan-Meier method and log-rank test. Cox regression (Proportional hazard model) was adopted for multivariate analysis of prognostic factors. Differences were considered statistically significant when *p* was less than 0.05.

## 5. Conclusions

Our data offer the convincing evidence for the first time that the combined miR-340 downregulation and ROCK1 upregulation may be linked to tumor progression and adverse prognosis in pediatric osteosarcoma. It should be further studied as a prospective biomarker in osteosarcoma.

## Figures and Tables

**Table 1.** Association of miR-340 and ROCK1 expression with clinicopathological features of pediatric osteosarcoma.

Clinicopathological features	No. of cases	miR-340-low (*n*, %)	*p*	ROCK1-high (*n*, %)	*p*	miR-340-low/ROCK1-high (*n*, %)	*p*
**Age**							

<13	52	28 (53.85)	NS	30 (57.69)	NS	24 (46.15)	NS
≥13	40	22 (55.00)		22 (55.00)		18 (45.00)	
**Gender**							

Male	56	32 (57.14)	NS	32 (57.14)	NS	25 (44.64)	NS
Female	36	18 (50.00)		20 (55.56)		17 (47.22)	
**Tumor size (cm)**							

>8	50	32 (64.00)	0.02	30 (60.00)	NS	25 (50.00)	NS
≤8	42	18 (42.86)		22 (52.38)		17 (40.48)	
**Localization of the primary tumor**							

Femur	53	30 (56.60)	NS	32 (60.38)	NS	27 (50.94)	NS
Tibia	25	15 (60.00)		15 (60.00)		10 (40.00)	
Humeral bone	9	3 (33.33)		3 (33.33)		3 (33.33)	
Other	5	2 (40.00)		2 (40.00)		2 (40.00)	
**Pathological facture**							

Present	10	6 (60.00)	NS	6 (60.00)	NS	4 (40.00)	NS
Absent	82	44 (53.66)		46 (56.10)		38 (46.34)	
**Subtype of osteosarcoma**							

Conventional	38	20 (52.63)	NS	22 (57.89)	NS	16 (42.11)	NS
Non-conventional	54	30 (55.56)		30 (55.56)		26 (48.15)	
**Chemotherapy**							

ADM/DDP	58	32 (55.17)	NS	32 (55.17)	NS	26 (44.83)	NS
ADM/MTX	34	18 (52.94)		20 (58.82)		16 (47.06)	
**Metastasis**							

Present	31	25 (80.65)	0.00	25 (80.65)	0.00	24 (77.42)	<0.001
Absent	61	25 (40.98)	1	27 (44.26)	1	18 (29.51)	
**Response to pre-operative chemotherapy**							
Good	50	32 (64.00)	0.00	34 (68.00)	0.00	28 (56.00)	0.002
Poor	42	18 (42.86)	2	18 (42.86)	2	14 (33.33)	

**Table 2.** Multivariate survival analysis of overall survival and progression-free survival in 92 patients with pediatric osteosarcoma.

Variables	Overall survival			Progression-free survival		

	RR	95% CI	*p*	RR	95% CI	*p*
miR-340 expression	6.2	1.4–13.9	0.006	4.5	1.0–9.2	0.01
ROCK1 expression	5.8	1.2–12.6	0.008	3.2	1.0–8.8	0.02
miR-340/ROCK1 expression	7.6	1.5–16.2	0.002	6.5	1.0–8.2	0.005
Tumor size	3.5	1.0–8.4	0.01	2.9	0.8–7.6	0.02
Metastasis status	4.2	1.6–10.3	0.006	3.9	1.3–9.6	0.008
Response to pre-operative chemotherapy	2.5	0.9–7.3	0.02	2.1	0.6–6.6	0.03
